# Cognitive Impairment in Acute Heart Failure: Narrative Review

**DOI:** 10.3390/jcdd8120184

**Published:** 2021-12-14

**Authors:** Ioannis Ventoulis, Angelos Arfaras-Melainis, John Parissis, Eftihia Polyzogopoulou

**Affiliations:** 1 Department of Occupational Therapy, University of Western Macedonia, 50200 Ptolemaida, Greece; 2 Heart Failure Unit and University Clinic of Emergency Medicine, Attikon University Hospital, National and Kapodistrian University of Athens Medical School, 12462 Athens, Greece; iparisis@med.uoa.gr (J.P.); effiepol@med.uoa.gr (E.P.); 3 Jacobi Medical Center, Albert Einstein College of Medicine, Bronx, NY 10461, USA

**Keywords:** acute heart failure, cognitive impairment, cardiocerebral syndrome, neurocognitive disorder, pathophysiological mechanisms, epidemiology, screening tools

## Abstract

Cognitive impairment (CI) represents a common but often veiled comorbidity in patients with acute heart failure (AHF) that deserves more clinical attention. In the AHF setting, it manifests as varying degrees of deficits in one or more cognitive domains across a wide spectrum ranging from mild CI to severe global neurocognitive disorder. On the basis of the significant negative implications of CI on quality of life and its overwhelming association with poor outcomes, there is a compelling need for establishment of detailed consensus guidelines on cognitive screening methods to be systematically implemented in the population of patients with heart failure (HF). Since limited attention has been drawn exclusively on the field of CI in AHF thus far, the present narrative review aims to shed further light on the topic. The underlying pathophysiological mechanisms of CI in AHF remain poorly understood and seem to be multifactorial. Different pathophysiological pathways may come into play, depending on the clinical phenotype of AHF. There is some evidence that cognitive decline closely follows the perturbations incurred across the long-term disease trajectory of HF, both along the time course of stable chronic HF as well as during episodes of HF exacerbation. CI in AHF remains a rather under recognized scientific field that poses many challenges, since there are still many unresolved issues regarding cognitive changes in patients hospitalized with AHF that need to be thoroughly addressed.

## 1. Introduction

Acute heart failure (AHF) is a heterogeneous clinical syndrome characterized by abrupt onset or gradual deterioration of symptoms and signs of heart failure (HF), in contradiction to chronic HF which represents a state of a much more gradual onset of symptoms or an already established diagnosis of HF [1]. In most cases, AHF manifests as acute decompensated chronic HF and less frequently as de novo HF. On clinical grounds, four distinct phenotypes of AHF can be recognized based on the presence or absence of congestion and peripheral hypoperfusion, namely, warm and wet, cold and wet, warm and dry, and cold and dry [2,3]. AHF remains one of the most common causes of hospital admission in western societies accompanied by high readmission rates and accounting for considerable in-hospital and post-discharge mortality [3,4,5,6]. It constitutes a tremendous and growing public health issue which imposes a substantial economic burden on the healthcare systems worldwide [7,8,9].

AHF can lead to dysfunction, injury, and eventually failure of many vital organs, which in turn contributes to further increase in the observed mortality. The brain represents one of the target organs involved in this process [10]. The heart and the brain are closely linked and interact bidirectionally through numerous feedback pathways [11]. Owing to the complex pathophysiological interplay between the heart and the brain, the notion of the cardiocerebral syndrome in HF has emanated, along the lines of the cardiorenal and the cardiohepatic syndrome [12].

In fact, the close interaction between the heart and the brain has long been recognized, dating back to 1977 where the term ‘’cardiogenic dementia’’ was introduced to describe the cognitive deterioration observed in patients with heart disease [13]. Nowadays, cognitive impairment (CI) is a well-established entity among patients with HF, encountered across a wide range of clinical presentations, that is both acute and stable chronic HF with either reduced or preserved ejection fraction [14]. CI is an inclusive term used to describe any kind of deficit in one or more cognitive domains, the extent of which cannot be attributed to the expected normal decline due to aging. In the AHF setting, a wide spectrum of CI can be encountered, spanning from mild CI to severe global neurocognitive disorder [14,15,16,17].

The negative consequences of CI cannot be overstated, bearing in mind that CI has been linked to poor medical adherence [18], low abidance to recommended lifestyle behaviors [19], and deficits in self-care [20]. Even mild CI may have a significant negative impact on quality of life by affecting important aspects of everyday living and by interfering with self-care management, which is considered a crucial component for the successful treatment of HF [21,22,23]. Moreover, poor self-care has been shown to be an independent risk factor for cardiac events, HF hospitalizations, and increased length of hospital stay [24]. More importantly, in HF patients, CI has been associated with an increased risk of cardiovascular events [25]. In hospitalized patients with AHF, CI has proven to be an independent prognostic marker of in-hospital mortality, prompting approximately a 5-fold increase in mortality [26].

The majority of the research studies examining the scope of CI in HF have included stable HF patients, while the reviews on the topic have made no discrimination between chronic stable and acute HF. Studies dealing with CI in AHF are rather limited. Here, we focus on the association between AHF and CI, review the screening approach towards HF-induced brain injury, outline aspects of epidemiology, explore potential pathogenic mechanisms in AHF that may underlie and predispose to the development of CI, describe the dynamic changes of CI during an episode of AHF, highlight the role of brain imaging modalities in the assessment of CI, and propose a therapeutic approach.

## 2. Cognitive Screening Tools

During the application of a cognitive screening test, the patient is requested to carry out a series of tasks that assess one or more cognitive domains [27]. Commonly evaluated cognitive domains include memory (immediate and delayed, episodic and semantic), attention/concentration, working memory, visuospatial/constructional ability, executive function, perceptual motor function/psychomotor speed, learning, speech/language, orientation, and social cognition [27,28,29,30,31]. A vast array of brief screening tests for CI is available for use in different clinical settings. These include the Mini Mental State Examination (MMSE), the Montreal Cognitive Assessment (MoCA), the Mini-Cog Test, the Clock Drawing Test (CDT), the Abbreviated Mental Test (AMT), the Short Portable Mental Status Questionnaire (SPMSQ), the St. Louis University Mental Status Examination (SLUMS), the Memory Impairment Screen (MIS)/ MIS by Telephone (MIS-T), the Free and Cued Selective Reminding Test, the 7-Minute Screen (7MS), the Telephone Instrument for Cognitive Status (TICS), the Informant Questionnaire on Cognitive Decline in the Elderly (IQCODE), the 8-Item Informant Interview (AD8), the Functional Activities Questionnaire (FAQ), and others [27]. It has to be emphasized that the aforementioned cognitive tests serve as screening tools and are not intended for diagnostic purposes. Once a screening test turns out positive, further clinical assessment and comprehensive neuropsychological evaluation is warranted to formally confirm the diagnosis of CI [27,32]. Nevertheless, one should always keep in mind that when assessing cognitive status, a number of possible confounding factors should be taken into account, such as age, mood disorders, education, employment status, ethnicity, linguistic diversity, and cultural and socioeconomical background [33,34,35,36]. Subsequently, neurocognitive test batteries, even more so cognitive screening tests, need to compensate for possible confounders in order to be reliable and applicable in an evergrowing cross-cultural diverse population.

In HF studies, a variety of screening tools for CI have been utilized, none of which have been specifically devised for HF patients. The same screening tools have been used interchangeably in all HF clinical settings, that is, both inpatients and outpatients and both stable and acute HF. The most widely used screening instruments in HF literature are the MMSE and the MoCA [30,31,37,38]. Yet, the need for even briefer cognitive screening tests to be applied in a hectic clinical setting led to the development of the Mini-Cog [39,40] and the MoCA 5 min protocol (Mini-MoCA) [41,42]. Some of the most common and brief screening tools for the assessment of CI in HF are illustrated in Table 1.

The MMSE is an instrument measuring global cognition, as it covers several cognitive domains, such as orientation, memory (registration and recall), attention and calculation, language, and visuospatial/constructional skills. It consists of eleven questions or tasks and is based on a scoring system ranging from 0 to 30, with higher scores indicating better cognitive performance. It can be easily applied by lay personnel with very little training and requires about 5–10 min to administer [43]. Its performance is however confounded by age, educational level, literacy status, verbal IQ level, and cultural context [35,44,45,46,47]. It has been also criticized due to its reduced sensitivity to detect mild CI [35,47,48] as well as its limited ability to identify alterations in cognition relating to frontal subcortical executive functions and right hemispheric lesions [35,47,49].

The MoCA is a cognitive screening instrument originally designed to detect mild CI. It assesses eight cognitive domains, namely, attention, concentration, working memory, short-term memory (delayed recall), executive function, language, visuospatial ability, and orientation. It utilizes a scoring system spanning from 0 to 30 and requires approximately 10 min to complete. The higher scores are suggestive of better global cognition. To allow for discrepancies in the educational level, the total final score is adjusted by adding 1 point for those with 12 years of formal education or less [50]. A basic feature of the MoCA is that it incorporates an explicit component of executive function assessment (modified Trail making B task, phonemic fluency task, similarities task through two-item verbal abstraction), which the MMSE lacks [50,51]. However, it has a relatively poor specificity, which renders it of limited use in clinical settings characterized by low base rates of mild CI [52,53].

In head-to-head comparison studies examining the performance of MMSE and MoCA in both HF outpatients [36] and inpatients [54], MoCA outperformed MMSE in terms of identifying early cognitive changes indicative of subtle CI. Subsequently, Hawkins et al. examined the performance of MoCA and MMSE against a comprehensive neuropsychological battery of tests, which assess attention, executive function, memory, and visuospatial ability, in a population of 106 HF outpatients aged 50–85 years. They observed that a MoCA cut-off of <25 yielded 64% sensitivity and 66% specificity, while a cut-off of <28 for the MMSE attained 70% sensitivity and 66% specificity. They concluded that application of both tests led to correct identification of the majority of patients with and without multi-domain CI, albeit both of them failed to properly classify approximately one third of the HF patients [55].

The magnitude and clinical consequences of CI in HF have long been appreciated, considering the fact that the Heart Failure Society of America (HFSA) incorporated in their 2010 practice guidelines the general recommendation to assess patients’ cognitive status and provide additional support in case of CI as part of disease management [56]. The 2016 ESC (European Society of Cardiology) guidelines for the diagnosis and treatment of acute and chronic HF moved a step forward by stating that ‘’Cognitive function can be assessed using the Mini-Mental State Examination or the Montreal cognitive assessment’’, while encouraging patient’s support by a multidisciplinary HF team including a specialist in neurocognitive disorders, along with family and caregivers, in order to optimize HF management and facilitate self-care [2].

To date, there is no disease-specific cognitive measure tool for identifying CI in HF patients. Virtues of an ideal screening measure for CI would be high sensitivity and specificity combined with brevity, reliability, simplicity, ease of administration in routine clinical practice, and ability to capture even mild CI affecting either a single or multiple cognitive domains. Although the most suitable screening tool for CI with optimal sensitivity and specificity, validated in HF populations and tailored for use in clinical practice is yet to be identified, it is imperative for clinicians to be aware of the high prevalence of CI in AHF and its implications and systematically screen for cognitive deficits.

## 3. Epidemiology

The reported prevalence of CI in AHF varies considerably. Figures ranging from 16% to 80% have been reported in the literature. This observed wide fluctuation in prevalence is attributed to many factors, such as varying definitions of CI, different screening tests deployed, disparate thresholds applied, non-homogeneity of the HF population studied (in terms of age, severity, and type of HF), sample sizes and diverse study design characteristics.

Early reports indicated that the prevalence of CI (determined by MMSE scores <24) in 50 patients ≥60 years old with decompensated HF was 54%, while almost three thirds of the patients seemed to experience signs of CI when a cut-off of CAMCOG score <80 was applied [57]. At the same time, data from an Italian multicenter survey, having enrolled more than 1100 patients admitted to the hospital with HF, revealed that CI, assessed by the Hodkinson Abbreviated Mental Test, was evident in 35% of the patients [26,58]. Later on, Debette et al. reported that some degree of CI was observed in two thirds of the overall population (83) of patients with decompensated HF included in their study. They showed that 61% of them had mild CI (defined as MMSE score ≤28 or ≤26 depending on their educational level), while 31% exhibited overt CI (defined as MMSE score ≤24) [59].

Meanwhile, in a prospective cohort study of 282 patients (mean age 80 years) hospitalized for AHF, CI was present in 46.8% of the overall sample with 25.2% meeting criteria for mild CI (MMSE score 21–24) and 21.6% for moderate–severe CI (MMSE score ≤20) [60]. Similar rates (45%) of at least mild CI (MoCA ≤ 22) were recorded by an Australian study following 565 patients with decompensated HF and a median age of 74 years [61], while the Vanderbilt Inpatient Cohort Study (VICS) reported a comparable degree (53%) of any level of CI (defined as an SPMSQ score ≥1) in 883 patients with AHF and a median age of 60 years [62]. Furthermore, in a study examining the effect of CI on 30-day readmission rates in a population of 241 elderly hospitalized patients (>70 years old), 121 of whom were admitted for HF as the primary diagnosis, it was found that 67.7% of the HF patients had CI defined as Mini-Cog scores of less than 4 [63]. In contrast, the first study to have used the Mini-Cog in 720 patients hospitalized for HF reported a 23% prevalence of CI by using a different cut-off score of ≤2 [39].

A much higher prevalence of CI (79%) in at least one cognitive domain was reported by Hajduk et al. in a cohort of 577 older (average age 71 years) patients with decompensated HF, who were evaluated in three cognitive domains with the use of standardized measures. Specifically, deficits in memory, mental processing speed, and executive function were noted in 33.3%, 40%, and 56% of the HF patients, respectively [64]. An equally high prevalence (80%) of CI was noted in at least one of the three cognitive domains (memory, executive function, processing speed) assessed in a study of 744 patients with acute decompensated HF and a mean age of 72 years. In particular, 32% of the patients demonstrated CI in only one domain, 31% in two domains, and 17% in three domains. Patients exhibited primarily deficits in executive function (59%) and in processing speed (51%) and to a lesser extent in memory (35%) [65].

Recent studies still continue to show discrepancy in the reported rates of CI prevalence. As a matter of fact, a Swedish prospective cohort study including 281 patients hospitalized for HF with an average age of 74 years reported that 29% of them displayed signs of CI based on MoCA scores <23 [66]. Even more so, data from the RICA registry indicated that, amongst 3845 patients (mean age 79 years) hospitalized for decompensated HF, 16% had significant CI (14% moderate and 2% severe CI). In this study, cognitive status was assessed by means of the Short Portable Mental Status Questionnaire (SPMSQ) and the patients were categorized into three groups based on their performance on the particular test: 0–3 errors were considered as no CI or mild CI, 4–7 as moderate CI, and 8–10 as severe CI. By using these cut-offs, there was no discrimination between intact cognition and mild CI and only the prevalence of moderate and severe CI was determined, hence the low prevalence reported by the investigators [67]. On the other hand, the REHAB-HF trial recorded a CI prevalence of 78% among 198 older (≥60 years old) hospitalized patients with acute decompensated HF when a MoCA score <26 was used. Of note, only 2% of these patients were clinically recognized as cognitively impaired [68].

Regardless of the conflicting estimates of its prevalence, CI represents a veiled comorbidity in patients with AHF that deserves more clinical attention, as it is intertwined with significant clinical ramifications and poor health outcomes.

## 4. Pathophysiology

The pathophysiology behind the induced brain injury in AHF still awaits to be elucidated as the underlying mechanisms are not well understood and seem to be multifactorial. Different pathophysiological mechanisms responsible for the observed brain changes in AHF could be proposed. Depending on the clinical phenotype of AHF, diverse pathophysiological pathways may come into play and exert synergistic effects, since no single mechanism can fully explain the cognitive changes that occur in AHF.

Under normal conditions, cerebral blood flow remains rather stable during wide fluctuations of the mean arterial blood pressure owing to inherent properties that the central nervous system possesses to activate and maintain elaborate vascular and neurohumoral mechanisms of autoregulation [69,70]. However, in HF states, these cerebral neuroprotective autoregulatory mechanisms are blunted, thus compromising cerebral blood flow and jeopardizing the brain’s integrity [71]. Cerebrovascular reactivity is also impaired in HF, as evidenced by the diminished response of the cerebral vasculature to hypercapneic conditions [72].

In the hypoperfused AHF states, the most plausible mechanism seems to be the decreased cerebral blood flow as a result of the reduced cardiac output, leading to inadequate blood supply to the brain and subsequent brain ischaemia [73,74,75]. The effect of the ischaemic assault to the brain is further magnified by the combination of transient mechanisms of hypoperfusion, whereby impaired cardiac performance manifested as low cardiac output acts synergistically with concurrent hypotension to yield a state of cardiogenic shock, which may be particularly detrimental to the brain. Furthermore, it is already known, based on the work of Gheorghiade et al., that systolic blood pressure at admission is an independent prognostic marker of morbidity and mortality in patients hospitalized with AHF and moreover low systolic blood pressure is associated with worse survival [76]. Zuccalà et al. reported that lower levels of systolic blood pressure were independently correlated with CI among older patients hospitalized with HF [77], while Hoth et al. found that systemic hypoperfusion indicated by low EF and low cardiac index was associated with deficits in several cognitive domains among HF patients [78]. Furthermore, other investigators reported a non-linear association between EF and cognitive performance, which became more pronounced at lower levels of EF and was further strengthened in the presence of concomitant hypotension [79]. Studies demonstrating significant cognitive improvement in post-heart transplant patients seem to confirm the hypothesis that systemic hypoperfusion due to impaired cardiac function is a principal causative factor of CI in HF [80,81]. Similarly, other studies noted that amelioration of cardiac function after cardiac resynchronization therapy was accompanied with improvements in cerebral blood flow and cognition [82,83]. Additionally, a study conducted in geriatric cardiac patients revealed that depressed cardiac output was associated with poorer cognitive performance in the domain of executive function [84]. Further support for the role of hypoperfusion in the pathophysiology of CI in HF is provided by imaging methods. Early reports exist even from the previous century stating that cerebral blood flow (estimated by the intravenous ^133^xenon injection method) is reduced in patients with severe HF [85]. Likewise, in patients with severe HF who were candidates for cardiac transplantation, cerebral blood flow estimated by single-photon emission computed tomography was decreased by approximately 30% at baseline, but interestingly it was restored within one month after heart transplantation [86]. A more recent study in HF patients, using transcranial Doppler ultrasound and brain magnetic resonance imaging, showed that reduced cerebral blood flow was associated with a greater degree of white matter hyperintensities and poorer cognitive performance [87].

Atrial fibrillation, which often complicates AHF, can further exacerbate CI. Indeed, atrial fibrillation can compromise cerebral perfusion by reducing systemic cardiac output via increased heart rate and reduced left ventricular systolic performance. It can also induce CI through alternative mechanisms, such as cerebral thromboembolism or cerebral microbleeds [88]. The phenomenon of the ischaemic insult to the brain may be further aggravated by the presence of multiple risk factors often found in HF patients and adding to the total cardiovascular risk burden. These risk factors include advanced age [89], arterial hypertension [90,91], diabetes mellitus [91,92], smoking [93], and obstructive sleep apnea [94], among others. Hyperhomocysteinaemia has also been associated with an increased risk for major neurocognitive disorders [95].

From a cellular point of view, cerebral hypoperfusion reduces the delivery of glucose and oxygen to the central nervous system. The deprivation of the brain’s main energy substrate and the resultant hypoxia lead to structural cellular changes of the neurons, alterations in the neuronal metabolism and disruption of the continuity of the blood–brain barrier. The energy shortage along with the established hypoxic conditions render the neurons susceptible to oxidative stress, inducing increased production of free radicals, ineffective protein handling, neuronal dysfunction, increased permeability of the blood–brain barrier, and oedema [96,97,98].

In the setting of hypertensive AHF, the elevated blood pressure results in an abrupt increase in cerebral blood flow, which cannot be effectively counterbalanced by appropriate constriction of the central nervous system’s vasculature due to the compromised autoregulatory mechanisms of the brain and the dysfunctional cerebrovascular reactivity [99,100,101]. Once the upper limit of cerebral autoregulation is exceeded, cerebral blood flow can no longer be maintained at a constant level and becomes directly proportional to the mean arterial pressure or, in other words, becomes pressure-passive [101]. When this breakthrough point is reached, segments of vasodilated vessels with alternating areas of vasoconstricted arterioles will be observed, since the loss of cerebrovascular autoregulation will lead to forced dilatation of certain arteriolar segments, while other portions of arterioles will remain physiologically constricted owing to the fact that the autoregulatory mechanism in these specific areas is still intact [101,102,103]. Accordingly, the unopposed abrupt rise in cerebral blood flow and pressure can disrupt the integrity of the endothelium of the brain vessels as well as the function of the blood–brain barrier, potentially leading to cerebral oedema [101,104].

In instances where congestion is the predominant clinical profile of AHF, it could be postulated that venous congestion in the brain may contribute to the development of CI through several different mechanisms. In detail, systemic congestion leads to increased central venous pressure which may then be transmitted backwards to the brain circulation, causing elevated cerebral venous pressure [105]. The phenomenon may be further aggravated by concomitant jugular venous reflux, which was found to be associated with CI when combined with high right atrial pressure [106]. The resultant retrograde increase in the cerebral venous pressure exerts adverse effects on cerebral haemodynamics by impeding cerebral venous return, promoting blood stasis in the brain, and elevating intracranial pressure, thus causing dysregulation of the cerebral perfusion pressure and cerebral blood flow. Moreover, increased cerebral venous pressure may account for the development of cerebral microhemorrhages of venous origin, the genesis of cerebral microinfarcts, the impaired function of the glymphatic system, alterations in the homeostasis of the cerebrospinal fluid, and the development of white matter hyperintensities [105]. In an experimental model of increased cerebral venous pressure, cerebral venous congestion was shown to disrupt the blood–brain barrier, impair blood flow, and promote neuroinflammation via activation of microglia and upregulation of proinflammatory mediators [107]. In other words, increased cerebral venous pressure induces a cascade of processes which have been implicated in the genesis of neuronal dysfunction and the development of CI.

Thromboembolism represents another mechanism through which HF can induce impaired cerebral perfusion. It can manifest either as ischaemic stroke or occult cerebral infarction. HF constitutes a hypercoagulable state, characterized by blood stasis, increased plasma viscosity, enhanced platelet aggregation, increased production of thrombogenic factors, reduced fibrinolysis, and impaired endothelial function. The compromised cardiac performance, in concert with the deranged haemorheological conditions that prevail in HF, promote thrombus formation through activation of the coagulation cascade, ultimately leading to cerebral infarction [108,109].

Apart from the aforementioned haemodynamic mechanisms, several other factors could also contribute to the development of CI in AHF. It is widely accepted that AHF is characterized by a state of excessive activation of the sympathetic nervous system and imbalance of the neurohormonal axis with overproduction of catecholamines and cortisol. Concordantly, there is an exaggerated expression of inflammatory mediators [10]. The undue activation of the neurohormonal system together with the overflow of proinflammatory cytokines exert deleterious effects on the neuronal cells, leading to an energy crisis and metabolic derangement of the neurons [98,110,111]. At the same time, a neurotoxic cascade is triggered, effectuating alterations in the cerebral beta-amyloid metabolism, which in turn generate additive effects by causing further brain insult [98,112].

The role of nutritional deficiencies has also been put forward as a potential mechanism for CI in HF, taking into account the fact that HF commonly induces a state characterized by insufficiency of nutrients via malabsorption, dietary restrictions, and use of certain medications (e.g., diuretics) [113]. As a matter of fact, thiamine deficiency induces oxidative stress and selective neuronal loss, while it has been linked to brain atrophy and white matter changes [114,115]. Along the same lines, iron deficiency, a very common HF comorbidity, has a negative impact on cognitive performance and should thus be treated accordingly [116]. Furthermore, low levels of serum albumin, which is a common finding in HF patients, especially in those with cachexia, have been associated with CI [58].

Moreover, depression seems to be tightly entangled with CI in HF patients and this correlation appears to be bidirectional and cumulative, as the presence of the former appears to deteriorate the latter and vice versa [33]. It has been reported that HF patients with depression have higher levels of proinflammatory cytokines, which have been implicated in the development of CI [117].

Lastly, iatrogenic factors should also not be overlooked. For instance, during treatment of hypertensive AHF, an inadvertent rapid decline in blood pressure could potentially provoke detrimental injury to the brain [118]. Moreover, in patients presenting with AHF, a plethora of factors can predispose the patient to an altered cognitive status, inducing even acute delirium. Such predisposing factors include deteriorating hypoxia [97,98], hypercapnia [119], electrolyte imbalances (e.g., hyponatraemia) [58,120], hypo- or hyper-glycaemia [121], anemia [122], renal [122] or hepatic failure [123], and drugs with anticholinergic properties [124].

## 5. Trajectory of CI over the Time Course of HF

Recently, a group of investigators attempted to estimate the prevalence of CI among 436 patients with incident HF (mean age 70 years) who were recruited in a prospective longitudinal cohort study (Regards study) evaluating participants at six-month intervals. Cognition was assessed with the Six-Item Screener (SIS) and scores ≤4 indicated CI. Interestingly, cognitive assessment was conducted prior to the incident HF diagnosis, thus reflecting cognition upon initial diagnosis of HF and minimizing any bias of clinical deterioration on cognition induced by the course of HF itself. The prevalence of CI in patients with incident HF (defined as the first hospitalization for HF without a previous history of HF) was 14.9%, being similar to that of a non-HF matched cohort (13.4%). Given the similar rates of CI prevalence between the two studied groups and taking into account the reported prevalence of CI in patients with already existing chronic HF (25–80%), the authors concluded that the highest burden of CI in HF develops after the HF diagnosis during the progression of the disease itself [125].

Once HF develops, further cognitive decline ensues, which parallels the duration and severity of HF. In line with this, Hammond et al. conducted a longitudinal analysis in the Cardiovascular Health Study concerning the time course of CI after newly diagnosed HF. The study included 4864 participants aged ≥65 years with a long follow-up. During a mean follow-up of 6.4 years, 496 patients with incident HF were identified. Cognitive performance was assessed annually using the 100-point Modified Mini-Mental State Examination (3MSE) and model-predicted trajectories of mean 3MSE scores were formulated for both study groups (no history of HF and after incident HF) and for different age groups. The investigators noted that the rate of cognitive decline was markedly faster after incident HF, compared to subjects with no HF, and the deterioration in cognition was much more pronounced at older ages. Remarkably, the rate of cognitive decline after incident HF was not significantly affected neither by atrial fibrillation status nor by EF category [126].

Likewise, secondary data analysis from the ACTIVE study indicated that patients with HF exhibit more rapid decline in cognitive function longitudinally (over a five-year period), albeit limited only to the cognitive domain of reasoning [127]. Along the same lines, a prospective longitudinal study on cognitive changes over a 10-year period of follow-up among 702 octogenarians with and without HF detected greater rates of cognitive decline in the field of episodic memory over time in those with HF [128].

On the other hand, in another longitudinal study, despite observing relative decline in global cognitive function of stable HF patients over a time period of two years compared to healthy controls, the investigators considered these cognitive changes to be subtle and not specific to HF when comparing them to patients with coronary artery disease [129]. Similarly, the TIME-CHF trial following 611 HF patients aged ≥60 years failed to detect significant changes in cognitive function in relation to changes in HF severity over a period of 18 months, since the prevalence of severe CI (defined as Hodkinson Abbreviated Mental Test score ≤7) remained rather stable over time. Yet, it is noteworthy that there was a significant rate of patient drop out with approximately two-thirds of those with severe CI at baseline being unavailable for follow-up assessment, which should be considered a major confounder [130]. On top of this, other researchers actually reported modest improvement in certain cognitive fields of patients with HF followed for a period of 12 months, highlighting that cognitive dysfunction in HF is not necessarily universal and could be potentially modifiable. However, there may have been a selection bias in this survey by not covering the full spectrum of HF patients (mild forms of HF patients who most probably abide by their medical regimens may have predominated) and by excluding patients with MMSE < 24. Moreover, the sample of HF patients was relatively small with an intermediate-term follow-up [131].

In acute decompensated HF it would be intriguing to assess the changes in cognitive function during the time window immediately after admission to the hospital (acute phase) up to the time point where full compensation has been achieved (compensated phase). This issue was addressed by Kindermann et al. in the CogImpair-HF study which examined the cognitive performance of 20 patients with decompensated HF before (within 48 h after admission) and after (14 ± 7 days) compensation in comparison to 20 patients with stable HF and 20 healthy controls. As expected, initially, patients with decompensated HF performed substantially worse than the other two groups, especially in the domains of memory, executive control, and processing speed, but once the compensated phase was reached, improvements were noted in their cognitive performance approaching the level of patients with stable HF [132].

**Figure 1:**
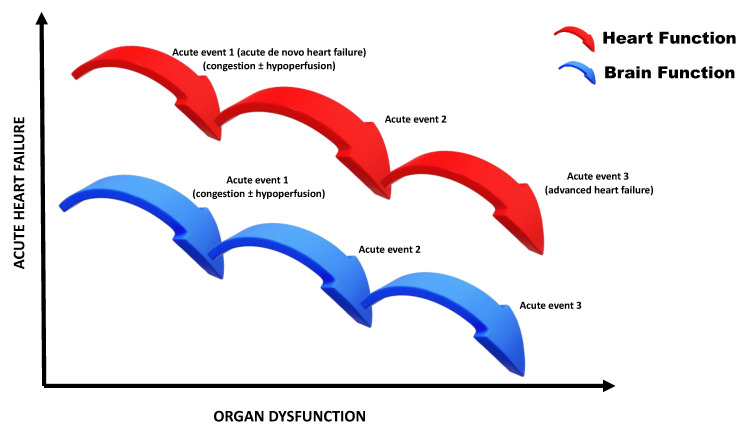
Progression of brain injury during the natural course of acutely decompensated heart failure.

Despite the paucity of longitudinal studies with an extensive follow-up, some evidence exists that cognitive decline closely pursues the perturbations incurred across the long-term disease trajectory of HF. The presumed longitudinal relationship between CI and HF is graphically depicted in Figure 1.

## 6. Role of Brain Imaging Modalities

Neuroimaging studies conducted in HF patients have documented structural abnormalities in several brain regions involved in cognition. Morphologic cerebral abnormalities, including cortical atrophy, ventricular enlargement, and cerebral infarcts, have been recognized long ago through brain magnetic resonance imaging (MRI) in HF patients with idiopathic dilated cardiomyopathy and have been related to worse cognitive performance [133]. More recent studies in HF patients have demonstrated regional volume reductions across certain brain cognitive areas, such as the hippocampus [134], the mammillary bodies [135], and the putamen [136]. Furthermore, another study showed reduced regional cortical thickness across multiple brain sites that control various high-level and autonomic functions. The observed findings reflect loss of neurons and supporting glial cells and exhibit lateralization, with the cortical thinning being more pronounced on the left side [137]. Almeida et al. found that HF patients demonstrated a higher burden of grey matter lesions, which were more extensive but with a similar pattern of topographic distribution, compared to patients with ischaemic heart disease [138].

With the use of 99 mTc-single-photon emission computed tomography (SPECT), regional cerebral blood flow was evaluated in HF patients and, when compared to controls, blood flow was found reduced in certain posterior cortical areas and was directly correlated to the degree of CI [139]. Subsequently, the same investigators supported that the regional cerebral blood flow abnormalities detected in HF patients resemble the regional patterns of functional brain deficits depicted in positron emission tomography (PET) imaging studies of glucose metabolism or amyloid deposition at the early stages of Alzheimer’s disease [140].

The aforementioned finding of regional cerebral hypoperfusion is in accordance with another study of HF patients reporting a significant reduction in cerebral blood flow, as estimated by 133Xe-SPECT, pre-transplant, which was consequently reversed following transplantation [86]. Accordingly, Alosco et al. recruited 69 HF patients who underwent brain MRI, transcranial Doppler ultrasonography of the middle cerebral artery, and cognitive assessment via the MMSE. They found that reduced cerebral blood flow was associated with greater white matter hyperintensities in older adults with HF [87]. In an attempt to further differentiate white matter injury into axonal versus myelin lesions, other investigators applied an MRI technique called diffusion tensor imaging to HF patients and reported both axonal and myelin injury, as evidenced by increased axial and radial diffusivity, respectively. The injury, comprising of either loss of axonal integrity or myelin breakdown or a combination of both, was evident along several fiber pathways in the brain, including cognitive circuits, such as fibers projecting to the limbic system, the thalamus, and the basal ganglia [141].

In order to relate neuroanatomical alterations in the grey and white matter with cerebral metabolic changes that occur in certain cognitive areas of the brain, additional studies are warranted in HF patients, ideally with the application of more sophisticated imaging modalities, such as 18F-FDG PET, amyloid PET imaging, functional MRI, and magnetic resonance spectroscopy [142,143,144,145].

## 7. Therapeutic Strategy

Once CI has been recognized, every effort should be made to limit its consequences by applying therapies that minimize or reverse brain injury. The main pillar of our therapeutic strategy should aim at preventing any subsequent CI, while effectively managing AHF. Of paramount importance is the prompt restoration of central haemodynamics, which will allow the stabilization of the patient with AHF. Any underlying comorbidities should also be addressed accordingly. In the AHF setting, any reversible cause of altered mental status should be systematically sought and immediately corrected [146].

**Figure 2:**
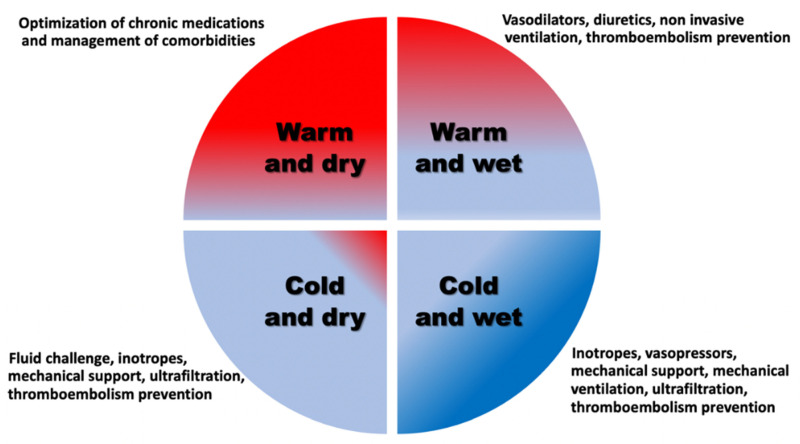
Protection of brain function through the optimal management of acute heart failure clinical profile.

It needs to be emphasized that there is no particular therapeutic intervention available for prevention and management of CI in AHF. In order to provide and ensure brain protection, the currently suggested approach is to abide by the recommendations provided by the clinical practice HF guidelines [1]. For the time being, applying optimal evidence-based medical treatment specifically tailored to each clinical profile of AHF, as per guidelines, should suffice. The proposed therapeutic approach, targeted at preventing CI and protecting the brain, depends on the AHF clinical phenotype and is presented in Figure 2.

## 8. Conclusions

While the exact pathophysiological mechanisms remain poorly understood and still need to be clarified, the evidence regarding the presence and burden of CI in AHF has been well established. Caution must be exercised given the fact that CI in AHF patients often goes unrecognized in the clinical setting. Most of the time, cognitive changes are subtle and may easily be overlooked by the clinician unless routinely scrutinized. Considering the overwhelming association of CI with poor outcomes, screening for CI has been recommended by HF guidelines, but unfortunately the scientific committees still omit to specify when and how to screen. Consequently, it is more than obvious that there is a compelling need for establishment of detailed consensus guidelines on cognitive screening methods to be systematically implemented in the population of patients with HF in the context of a more integrated approach.

Undoubtedly, more well-designed trials are needed to precisely delineate the longitudinal effects that HF exerts on cognitive function both over the time course of stable chronic HF as well as during episodes of HF exacerbation. There are still many unresolved issues regarding cognitive changes in patients hospitalized with AHF that need to be thoroughly addressed in the future. CI in AHF remains a rather undiscovered and under recognized scientific field that poses many challenges and awaits to be unraveled by future investigators.

## Figures and Tables

**Table 1:** Features of the most common and brief screening tools for the assessment of cognitive impairment in heart failure.

Screening Tool	Scoring System	Usual Cut-Off Point	Administration Time (min)	Comments
Mini Mental State Examination (MMSE) [35,43,44,45,46,47,48,49]	0–30	<24	5–10	■ Higher scores indicate better cognitive performance ■ Varying cut-offs have been used in different settings ■ Insufficient sensitivity for mild cognitive impairment ■ Fails to cover the domain of executive function ■ Affected by age, education, and cultural background
Montreal Cognitive Assessment (MoCA) [50,51,52,53]	0–30	<26	10	■ Higher scores indicate better cognitive performance ■ Lower cut-offs have been utilized when screening for mild CI in HF ■ Poor specificity ■ Covers executive function ■ Adjustment according to educational level
Mini-Cog Test [39,40]	Composite score: 5 3-item recall task: 0–3 CDT: normal (2) or abnormal (0)	≤2	3	■ Higher scores indicate better cognitive performance ■ Combination of Clock Drawing Test (CDT) with a simple three-item recall task: Patient is asked to repeat 3 unrelated words, then complete the clock drawing test and finally recall the initial 3 words ■ Not influenced by education or language ■ Does not require specialized training or equipment ■ CDT element is vulnerable to subjective interpretation by the examiner ■ Tests executive function and memory
MoCA 5-min protocol (Mini-MoCA) [41,42]	12	≤9	5	■ Higher scores indicate better cognitive performance ■ Different mini versions exist with varying scoring systems (the scores and cut-offs provided here have been proposed for HF patients) ■ Still awaits to be standardized and validated ■ Derived by extracting 3 or 4 subtests from MoCA (depending on the version) ■ Tests memory, executive function/language, orientation (±attention depending on the version) ■ Can be administered over the telephone
